# Use of Mature miRNA Strand Selection in miRNAs Families in Cervical Cancer Development

**DOI:** 10.3390/ijms18020407

**Published:** 2017-02-14

**Authors:** Angelica Judith Granados-López, José Luis Ruiz-Carrillo, Luis Steven Servín-González, José Luis Martínez-Rodríguez, Claudia Araceli Reyes-Estrada, Rosalinda Gutiérrez-Hernández, Jesús Adrián López

**Affiliations:** 1 Laboratorio de microRNAs, Unidad Académica de Ciencias Biológicas, Universidad Autónoma de Zacateacs, Av. Preparatoria S/N, Zacatecas 98066, Mexico; agranadosjudith@gmail.com (A.J.G.-L.); jocaru2012@gmail.com (J.L.R.-C.); joseluis_mtzrdz_qfb@hotmail.com (J.L.M.-R.); 2 Doctorado en Ciencias Básicas, Universidad Autónoma de Zacateacs, Av. Preparatoria S/N, Campus II, Zacatecas 98066, Mexico; 3 School of Life Sciences, Gibbert Hill Campus, University of Warwick, Coventry CV47AL, UK; L.Servin-Gonzalez@warwick.ac.uk; 4 Doctorado en Ciencias Básicas en la Especialidad en Farmacología Médica y Molecular de la Unidad Académica de Medicina Humana y Ciencias de la Salud de la Universidad Autónoma de Zacateacas, Campus Siglo XXI, Kilómetro 6, Ejido la Escondida, Zacatecas CP 98160, Mexico; c_reyes13@yahoo.com.mx (C.A.R.-E.); rosalindagh@hotmail.com (R.G.-H.)

**Keywords:** miRNA families, strand 5p, strand 3p, cervical cancer

## Abstract

Aberrant miRNA expression is well recognized as a cancer hallmark, nevertheless miRNA function and expression does not always correlate in patients tissues and cell lines studies. In addition to this issue, miRNA strand usage conduces to increased cell signaling pathways modulation diversifying cellular processes regulation. In cervical cancer, 20 miRNA families are involved in carcinogenesis induction and development to this moment. These families have 5p and 3p strands with different nucleotide (nt) chain sizes. In general, mature 5p strands are larger: two miRNAs of 24 nt, 24 miRNAs of 23 nt, 35 miRNAs of 22 nt and three miRNAs of 21 nt. On the other hand, the 3p strands lengths observed are: seven miRNAs of 23 nt, 50 miRNAs of 22 nt, six miRNAs of 21 nt and four miRNAs of 20 nt. Based on the analysis of the 20 miRNA families associated with cervical cancer, 67 3p strands and 65 5p strands are selected suggesting selectivity and specificity mechanisms regulating cell processes like proliferation, apoptosis, migration, invasion, metabolism and Warburg effect. The insight reviewed here could be used in the miRNA based therapy, diagnosis and prognosis approaches.

## 1. Introduction

Genes are classified in families based on three characteristics, their phylogenetic origin, sequence similitude and possible functional homology [1], sharing several mechanisms of regulation. Most families have evolutionary conservation implying an important biological function [1,2]. Members of different miRNA families have evolved to target a diverse set of transcripts by mechanisms including arm switching, seed shifts, insertions and nucleotide editing of mature transcripts, giving rise to different seed sequences and hence altered target specificities [3]. The members of the different miRNA families present overlapping mRNAs target and tissue specificity [4]. Interestingly, the miRNAs from a family do not express equally, neither do the 5p nor 3p strand of a pre-miRNA [2,5,6,7]. Gene regulation by miRNAs acquired a complexity achieved by several factors like the type of promoter that determines the regulation of mRNAs targets [8], RNA edition [9], RNA base addition [10,11], RNA slicing [12], and miRNA strand selection [13], among others. miRNA strand maturation is a complex process consisting of two cuts, sequence thermodynamic instability and protein interactions. The 5p strand is produced by the cut of the microprocessor (Drosha and DGCR8) [14] and other proteins that interact with this complex like, p68, p72, Smads, p53 and ERα. Other proteins directly interact with the structure and sequence of pri-miR and pre-miR, like hnRNP A1 and KSRP that bind to and increase pri-miRNA and pre-miRNA dicing in contrast to Lin 28 that inhibits the cuts [15]. The 3p strand is formed by Dicer dicing and its activity is favored by TRBP and PACT interaction. Protein post-translational modifications contribute to miRNA cropping. Phosphorylation of TRBP via MAPK increases enzymatic function of Dicer as well as hydroxylation by type I collagen prolyl-4-hydroxylase stabilizes Ago 2 [16]. Thermodynamic strand selection obeys to GC and AU content in 5′ of 5p and 3p strand, considering that a high content of AU in the 5′ of 3p strand is going to be selected over 5p strand and vice versa. Protein participation in miRNA strand selection could override thermodynamic properties of miRNA sequence. However, the combination of both mechanisms is evolutionarily conserved and used to select miRNA strands conducing to gene expression regulation. The selection of miRNA strand is dependent of cell type, tissue and stimulus [14]. Sequence conservation and RNA binding-proteins evolved together to achieve a final fine-tuning gene regulation in different cells and tissues. Cervical cancer possesses particular cell types with specific differentiation patterns as a result of a special and unique gene expression. Previously, we analyzed 53 miRNAs reported in the literature associated with cervical cancer progression based on constant expression reported [17] and from these genes we further studied the families of miRNAs and the cell signaling pathways associated with 21 miRNA families grouped in 20 clusters [18] without noticing strand participation. Many of the studies reviewed did not specify the mature strand used in the investigation, while the star strand is almost never studied. Conventionally, mature miRNA strand performing silencing is recognized as guide strand and the other one as star (*) strand or passenger strand, which is usually degraded. However, guide and star strands have hundreds of potential targets and every family member has a particular mature miRNA expression that determines differential or complementary functions that regulate cell processes. In this review, we focused on strand selection of 20 families of miRNAs involved in cervical cancer based on expression and known function with the objective to uncover the importance of strand selection in cervical cancer. We investigated mature miRNA used in the studies reported and we observed a constant expression of miRNA guide strand from some members of miRNA families, however, for miR-10a, miR-15a, miR-16, and miR-200a, expression of both guide and star strands was noticed. Importantly, miRNA star strand expression of the rest of members of the 20 families associated in cervical cancer is currently unknown. Additionally, we performed miRNA families sequences alignments to analyze sequence conservation and nt chain length observing a great variation in sequence homology and nt chain size among families members supporting differential target gene regulation.

## 2. Anti-Oncogenic miR-1 Family Participates in Cervical Cancer

**Figure 1:**
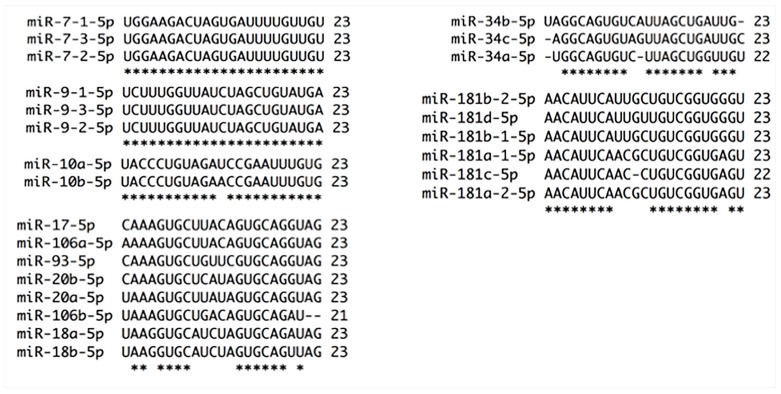
Alignments of miRNAs families by strand 5p selection and nt chain sizes. Six miRNA families were chosen by strand selection and nt chain size. Almost all members of these families are 23 nt in length, except miR-34a, and share high sequence conservation. miR-7 and miR-9 families have 100% of homology, miR-10 family varies only in 1 nt, miR-181 family is dissimilar in 3 nt, miR-17 family has five different nt, and miR-34 family presents six dissimilar nt. * at the bottom of the alignment indicates nt homology between family members.

**Figure 2:**
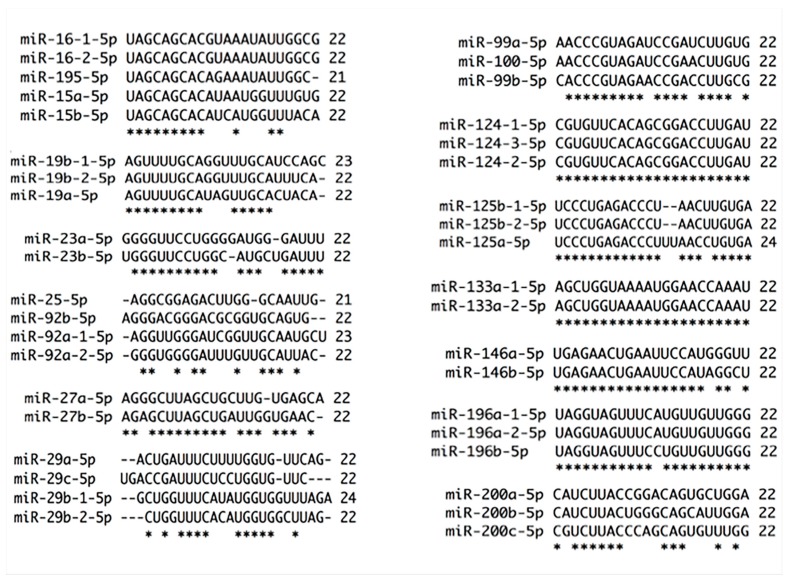
Alignments of miRNAs families by 5p strand selection and nt chain sizes. Thirteen miRNA families were chosen by strand and nt chain sizes. Almost all members of these families are 22 nt in length, except miR-195 (21 nt), miR-19b-1 (23 nt), miR-25 (21 nt), miR-92a-1 (23 nt), miR-29b-1 (24 nt), and miR-125a (24 nt). Of 13 miRNA families analyzed, only five use 5p arm as guide strand. miR-15 family shares 54.45%, miR-99 family 81.81%, miR-125 family 86.36%, miR-146 family 90.90% and miR-196 95.45% strand conservation. The rest of the families use the strand 3p, therefore, they show less 5p strand conservation. miR-19 family has 63.63 %, miR-23 family 77.27%, miR-25 family 45.45%, miR-27 family 72.72%, miR-29 family 45.54 %, miR-124 100%, miR-133 100% and miR-200 family 72.72% conservation. * at the bottom of the alignment indicates nt homology between family members.

**Figure 3:**
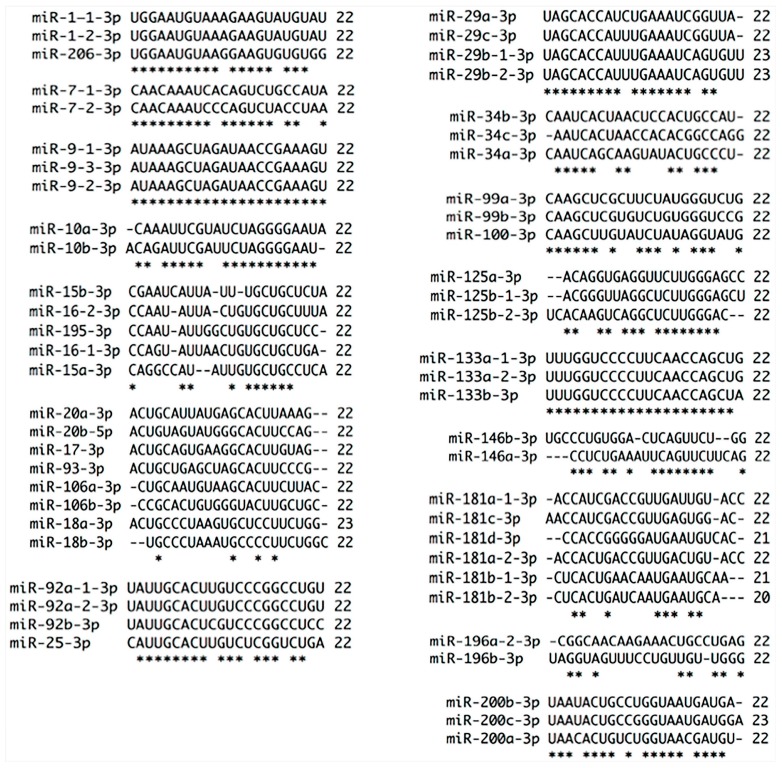
Alignments of miRNAs families by 3p strand selection and nt chain sizes. Sixteen miRNA families were chosen by miRNA strand and nt chain sizes. Almost all members of these families are 22 nt in length except miR-29b-1, miR-29b-2 (23 nt), miR-181b-1 (21 nt), miR-181b-2 (20 nt) and miR-200c (23 nt). Of 16 families analyzed, only five use 3p arm as guide strand. miR-1 family shares 81.81%, miR-25 family 72.72%, miR-29 family 77.27%, miR-133 family 100% and miR-200 72.72% strand conservation. The rest of the families use the strand 5p, therefore, they show less 3p strand conservation. miR-7 family has 81.81%, miR-9 family 100%, miR-10 family 77.27%, miR-15 family 45.45%, miR-34 family 54.54 %, miR-99 family 68.18%, miR-125 family 59.09%, miR-146 family 54.54% and miR-196 family 36.36% strand conservation. * at the bottom of the alignment indicates nt homology between family members.

miRNA-1 (miR-1) family is formed by miR-1-1, miR-1-2 and miR-206; nevertheless, it should be noted that miR-1-1 and miR-1-2 originate the same mature sequence, miR-1-3p. It is important to remark this particular issue because the pri-miR and pre-miR of miR-1 sequence and chromosome localization are different, therefore, biogenesis regulation could be singular for each one. miR-1-1 and miR-1-2 are localized in chromosome 20 and 18, respectively, while miR-206 is found in chromosome 6 [19,20]. Interestingly, despite sequence similarity, it has been shown that 5p strand of miR-1-2 and miR-206 is not expressed therefore it does not appear in Figure 1 or Figure 2, in contrast to 3p strand of miR-1 (Figure 3). miR-1-1-3p and miR-1-2-3p have 100% sequence similarity while miR-206 has four different bases (Figure 3). Identification of 5p strand of miR-1-1 has been reported in 10 experiments achieving 37 reads while 3p strand has more than 759,000 reads [20]. miR-1-3p has been shown down-regulated in cervical cancer versus normal tissue [17] and its overexpression diminishes proliferation, tumor growth and promotes apoptosis by glucose-6-phosphate dehydrogenase (G6PD) down-regulation [21] functioning as anti-oncomiR (Table 1).

## 3. miR-7 Family Participates in Cervical Cancer

miR-7 family is formed by miR-7-1, miR-7-2 and miR-7-3 localized in chromosomes 9, 15 and 19, respectively. MiR-7-3-3p arm has not been annotated [20] (Figure 3). The 5p arms share 100% similarity between family members, whereas 3p strands differ in four nucleotides between them (Figure 1). The difference between 3p strands of miR-7 family could lead to a distinct type of mature miRNA biogenesis selection conducing to specific target mRNA regulation that should be evaluated in cervical cancer progression. It is well known that a single nucleotide change in miRNAs could change target specificity [9]. Mature 5p strand of miR-7 is down-regulated in cervical cancer versus normal tissue and its overexpression inhibits the Focal Adhesion Kinase (FAK) and X-Linked Inhibitor of Apoptosis (XIAP) oncoproteins formation inducing apoptosis, inhibiting proliferation, migration and invasion [22,23]. On the contrary, two independent works show this miRNA as up-regulated in tumors versus normal tissue [24,25], suggesting that it functions like an oncogene. However, its function and expression do not correlate with cervical cancer progression [22,23]. In fact, miR-7 overexpression could be a signal to halt carcinogenesis process that is overridden by signals inducing cervical cancer development.

## 4. Oncogenic miR-9 Family Participates in Cervical Cancer

miR-9, another microRNA implicated in carcinogenesis [17], is localized in chromosomes 1, 5 and 15 and generates transcripts for miRNA-9-1, miR-9-2 and miR-9-3, respectively. Each strand from the three members can be loaded on RNA-Induced Silencing Complex (RISC) machinery based on strands homology and conservation (Figure 1 and Figure 3), suggesting a conserved and vast variety of mRNA targets involved in cancer progression. However, 5p strand abundance is superior more than 174 times to 3p strand [20]. In addition, 5p strand has been detected in at least two independent works [26,27] while 3p strand has not been detected in cervical cancer. miR-9 increase in squamous cervical cancer is associated with Human Papillomavuris E6 (HPV-E6) presence [28,29], however, in adenocarcinoma it has been reported as down-regulated by hypermethylation and its overexpression diminishes proliferation and migration via inhibition of Jak/STAT3 signaling activity through 3′UTR CKAP2, HSPC159, IL-6 and TC10 regulation [30]. We observe discrepancy between squamous and adenocarcinoma miR-9 expression from cervix, however, it is important to note that different cells and tissues from the same organ have distinctive miRNA expression and probably function as well.

## 5. miR-10 Family Participates in Cervical Cancer

miRNAs deregulation correlates with oncogenic or anti-oncogenic function such as the case of miR-10 family that is composed of miR-10a and miR-10b localized in chromosomes 17 and 2, respectively [20,31]. Interestingly, the members of this family have shown opposite expression [32] (Table 1). Additionally, 5p and 3p strands of miR-10a have shown different expression [33] (Table 1). The seed sequence of miR-10a and miR-10b is identical, therefore it should be expected that they share similar transcript targets, however, they have different expression and function. miR-10a-5p shows a progressive increase through Steps 1 to 4 of carcinogenesis [17,32,34,35] and induces colony formation, migration and invasion in HeLa, C-33A and SiHa cells by down-regulation of Cell Adhesion Molecule L1 Like (CHL1) and Phosphatase and Tensin Homolog PTEN via 3′UTR [36,37]. miR-10b-5p has been shown decreased in cervical cancer [24,25] as well as in pre-malignant steps and its overexpression reduces proliferation and invasion via 3′UTR down-regulation of Homeobox A1 (HOXA1) [38]. It should be noted that 5p strand of miR-10a and miR-10b potentially regulate CHL1, PTEN and HOXA1 (Target Scan, mirnaorg.com and Diana-microt) based on 95.65% of similitude (Figure 1). 3p strand of miR-10a and -b share 77% of sequence similarity (Figure 3), therefore mRNAs targets variability should increase diversifying the cellular pathways regulated by these strands.

## 6. Oncogenic miR-15 Family Participates in Cervical Cancer

miR-15 family is composed of miR-15a, miR-15b, miR-16 and miR-195 [39], localized in different genomic context (Table 1), hence their expression in cervical cancer development could be attributable to similar gene regulation achieved by cellular advantages acquired during carcinogenesis. In a similar fashion to miR-10 family, miR-15 family shows contradictory effects in cervical cancer progression, while miR-15a, miR-15b and miR-16 are shown overexpressed, miR-195 is down-regulated from Step 2 to 4 of carcinogenesis [17,35,40]. miR-15 and miR-16 overexpression inhibit proliferation, G1-S cycle transition in HeLa cells attenuating mTOR and p70S6K phosphorylation by down-regulation of Rictor inhibiting p62 a selective substrate for autophagy-lysosome degradation and increasing LC3-II conversion an autophagy gene-associated [41]. Interestingly, miR-15a-3p has a contrary function to miR-15a-5p, since the 3p strand triggers caspase 3/7 activation and reduces HeLa cells viability through Bcl-XL via 3′UTR [42]. In relation to miR-16-1, it shows an inverse expression with Cyclin E1 mRNA in SiHa, HeLa and CasKi cells, however, luciferase and protein data is missing [43]. Furthermore, miR-16-2-3p has been shown up-regulated in serum pointing out the role of miR-16 in cervical carcinogenesis [40]. On the other hand, miR-195 is shown down-regulated in cervical cancer and its overexpression inhibits proliferation, G1-S transition, migration and invasion through Cyclin D1, Cyclin D2, MYB proto-oncogene, transcription factor (MYB) and Smad 3 down-regulation via 3′UTR [44,45,46,47].

Since mature miRNA sequences of members of miR-15 family have shown expression differences, the effects on targets are expected to be different. As can be seen in Figure 2, seed sequence of 5p strands is similar among family members, contrary to 3p strand that is totally different between them (Figure 3). However, in 3p strand, there are six conserved nt between family members raising the possibility to regulate several targets taking count that miRNAs regulate mRNAs independently of seed sequence [48]. One point of miRNA regulation is the strand selection, thereby, while the guide strand is incorporated to Ago protein to form miRISC complex, * strand is degraded as a natural biogenesis process. Nevertheless, for several miRNAs, both strands are incorporated into miRISC complex and strand selection depends on tissue, cell type and proteins present [49,50]. In this respect, which strand is selected under different types of stress, such as cancer progression, has not been studied.

## 7. Oncogenic miR-17 Family Participates in Cervical Cancer

The members of miR-17 family have also shown differential expression patterns (Table 1), a characteristic shared with several miRNA families. miR-17 family is integrated by miR-17, miR-18a, miR-18b, miR-20a, miR-20b, miR-93, miR-106a and miR-106b. Most of the members of this family differ in mature sequence by 10 nt [51] (Figure 1 and Figure 3), implying a diverse function on mRNA targets regulation. In general, miR-17 family 5p strands have five different bases. miR-20a-5p and miR-20b-5p have only one different nucleotide in mature sequence as well as miR-17-5p and miR-106a-5p. miR-93-5p is the most dissimilar within the members of this family presenting three different bases with miR-20a-5p and miR-20b-5p, four with miR-17-5p and five with miR-106a-5p (Figure 1). 3p strands of the members of this family have 14 nucleotides of dissimilarity. miR-20a and miR-20b have five different bases; and miR-17-3p has six, seven and eight nucleotides different from miR-20b-3p, miR-20a-3p and miR-93-3p, respectively. miR-106a-3p has 10 dissimilar nt from miR-93-3p, nine from miR-20b-3p, eight with miR-17-3p and eight with miR-20a-3p (Figure 3).

miR-17-5p has been shown down-regulated while miR-20a, miR-20b, miR-93, miR-106a and miR-106b are found up-regulated exhibiting important changes during cervical cancer progression [17,52]. miR-18a is up-regulated in HPV-related exosomes [53] and induces radiosensitivity in cells irradiated, reducing ATM and γ-H2AX proteins, triggering apoptosis by caspase 3 activation and PARP cleaved [54]. miR-20a-5p up-regulates Tankyrase 2 (TNKS2) and down-regulates Autophagy Related 7 (ATG7), an essential protein for autophagy and transport from cytoplasmic to vacuole and Metallopeptidase Inhibitor 2 (TIMP2), a natural protein inhibitor of the matrix metalloproteinases, in a sequence dependent form reducing apoptosis and inducing colony formation, migration, and invasion [55,56]. miR-93 is increased from Step 1 to 4, augmenting proliferation and decreasing apoptosis through down-regulation of RAB11 Family Interacting Protein 1 (RAB11FIP1), a protein involved in endocytic sorting, trafficking of proteins including integrin subunits and epidermal growth factor receptor (EGFR), and transport between the recycling endosome and the trans-Golgi network [57]. miR-106b is up-regulated in cervical cancer through TGF-β1 eliciting migration by down-regulation of Disabled Homolog 2, Mitogen-Responsive Phosphoprotein (Drosophila) (DAB2) via 3′UTR. DAB2 is a protein that binds to the SH3 domains of GRB2, an adaptor protein that couples tyrosine kinase receptors to SOS through its C-terminal proline-rich sequences, and may thus modulate growth factor/Ras pathways by competing with SOS for binding to GRB2 [58]. In accordance with Pictar, MIRDB, and microrna.org TNKS2, ATM, ATG7, TIMP2 and RAB11FIP1 are potentially regulated by miR-17-5p, miR-106a-5p, miR-106b-5p, miR-93-5p and miR-20b-5p. This assumption is made for these genes, however, it is important to take into account that every miRNA potentially regulates more than 100 genes; therefore, experimental validation is imperative.

## 8. miR-19 Family Participates in Cervical Cancer

**Figure 4:**
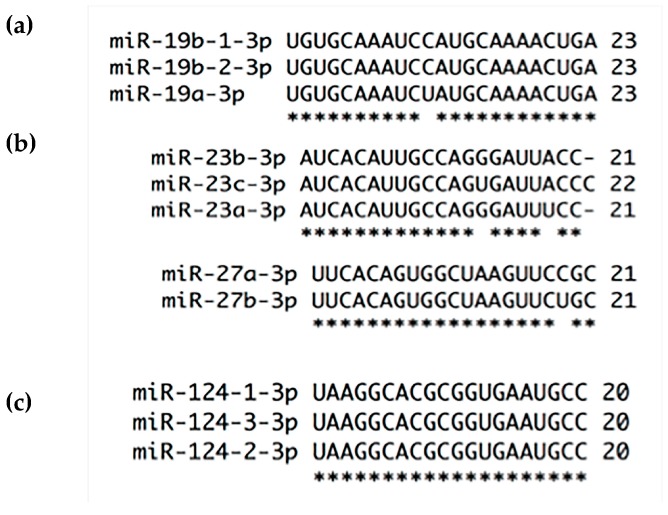
Alignments of miRNAs families by 3p strand used and nt chain sizes: (**a**) miR-19 family has 23 nt of length and share 100% of strand conservation; (**b**) miR-23 and miR-27 families are in general 21 nt in length except for miR-23c (22 nt). The former has 86.36% while miR-27 has 95.23% strand conservation; (**c**) miR-124 family shares 100% strand conservation and its members are 20 nt in size. * at the bottom of the alignment indicates nt homology between family members.

miR-19 family is composed of miR-19a, miR-19b-1, and miR-19b-2 [51]. Increased expression of miR-19a/b is involved in cervical carcinogenesis [17] probably by Cullin 5 (CUL5) down-regulation [59]. The 3p arm from both miRNAs are recognized as the guide strand [20] and, as can be observed in Figure 4, these strands only have one different nt between miR-19a and miR-19b in contrast with the 5p arms, which vary in more than 8 nt (Figure 2). Based on sequence similarities, it is possible that all members of this family could regulate CUL5 and several additional genes. Nevertheless, the * strand could open a vast set of new and important mRNAs targets.

## 9. Anti-Oncogenic miR-23 Family Participates in Cervical Cancer

miR-23 family is composed of miR-23a, miR-23b and miR-23c, the former is in chromosome 19, the middle in chromosome 9 and the last one in chromosome X [20]. The 3p strand is recognized as the guide strand and has less mature sequence difference than 5p strand. The 3p strands have 3 different nt between family members (Figure 4), while the 5p strands have five dissimilar nt and it is present in only two members (Figure 2). miR-23b, a member of this family, is involved in cervical carcinogenesis with a remarkable down-regulation during Steps 2 to 4 of the cervical multistep model of carcinogenesis that we recently proposed [17]. The promoter of miR-23b is methylated in cervical cancer [60] and has a consensus p53-binding site, therefore p53 diminish by the presence of HR-HPV16-E6 oncoprotein induces a down-regulation of miR-23b and an increased expression of uPA via 3′UTR mRNA binding [61]. This protein is a serine protease that degrades extracellular matrix regulated through Notch signaling, interestingly these two proteins are overexpressed in cervical cancer [17,18]. The 3p arm of the three members (a, b and c) of miR-23 family potentially regulate uPA mRNA (microrna.org, MIRDB and Target Scan).

## 10. Oncogenic miR-25 Family Participates in Cervical Cancer

miR-25 family is formed by miR-25, miR-92a-1, miR-92a-2, and miR-92b. The 3p strand of members of this family differs in mature sequence by 6 nt. miR-92a-1-3p and miR-92a-2-3p are equal while miR-92a-3p differs in 3 nt with miR-92b-3p (Figure 3). The 5p strand does not have a conserved seed sequence between the family members (Figure 2). However, this observation does not imply that these mature miRNAs do not regulate mRNAs individually. It has been addressed that the members of a same family regulate similar mRNA targets, however, differences in one nucleotide could change mRNA targets specificity and it can be observed that in some families the mature sequence varies in more than 4 nt. miR-25-3p and miR-92a are up-regulated in cervical cancer progression [62]. miR-25-3p overexpression suppresses tumor growth in mice and diminishes Semaphorin 4C (Sema 4c) via 3′UTR, Snail, and Vimentin while *E*-Cadherin increases suppressing epithelial–mesenchymal transition in cisplatin-resistant cells [63]. miR-92 has been shown to be increased in cervical cancer versus normal tissue [34,55,62], and its overexpression in SiHa and C-33A cells increases proliferation and migration while inhibition induces apoptosis as well as diminishes tumor growth [64]. miR-92a overexpression augments proliferation, G1-S transition and invasion by F-box and WD repeat domain-containing 7 (FBXW7) through 3′UTR [65].

## 11. Oncogenic miR-27 Family Participates in Cervical Cancer

miR-27 family is composed of miR-27a and miR-27b. miR-27a is an intergenic miRNA localized in chromosome 19 while miR-27b is an intronic miRNA located within the fourteenth intron of the human C9orf3 host gene in chromosome 9 [20]. The mature 3p arms of miR-27a and miR-27b differ in one nucleotide and share target mRNAs [66] (Figure 4), while the 5p arms have 5 nt of difference between them (Figure 2). Additionally, miR-27a and miR-27b have opposite expression patterns in the development of cervical carcinoma as the changes are recorded from Step 2 to Step 4 [17,67]. miR-27a binds to 3′UTR of beta-1,4-Galactosyltransferase III (B4GALT3) increasing protein synthesis. B4GALT3 is responsible for poly-*N*-acetyl lactosamine and is implicated in keratin sulfate/keratin metabolism and transport to Golgi and subsequent modification inducing migration and invasion, probably by increasing β1-integrin stability [68]. Two independent works [52,69] have shown an opposite expression for miR-27b compared to the report reviewed in Granados-López et al. [17]. Augmented expression of miR-27b elicits proliferation, G1-S transition, migration and invasion by reducing Peroxisome Proliferator Activated Receptor Gamma (PPAR-γ) and Cadherin 11 (CDH11) protein production via 3′UTR. PPAR-γ is a member of the nuclear receptor family of ligand-activated transcription factors that heterodimerize with the retinoic X receptor (RXR) to regulate gene expression while CDH11 is a calcium-dependent cell adhesion protein that preferentially interacts with itself in a homophilic manner in connected cells [52,69]. miR-27a and miR-27b could share several targets, however, their expression patterns differ in cervical cancer; consequently, target genes may be differentially regulated which indicates their relevance in cervical carcinogenesis.

## 12. Anti-Oncogenic miR-29 Family Participates in Cervical Cancer

Another family of miRNAs that shows important changes during Steps 1 to 4 of carcinogenesis is miR-29 family that is formed by miR-29a, -b and -c [17,70]. miR-29a has been shown reduced in serum of cervical cancer patients [71]. Interestingly, miR-29s have different subcellular localization while miR-29a and miR-29c are primary expressed in the cytoplasm, miR-29b is expressed in the nucleus [72]. The strand 5p has 14 dissimilar nt implying a great target plethora and differential mechanism of regulation. miR-29a-5p present seven different nt with miR-29b-1-5p, miR-29b-2-5p and miR-29c-5p, additionally, miR-29b-1-5p and miR-29b-2-5p have four different nt (Figure 2). miR-29 members have different effects on cell cycle indicating target specificity [73]. Target specificity could be explained by five dissimilar nt in the guide 3p strand in addition to strand longitude while miR-29-b has 23 nt miR-29a and miR-29c have 22 nt (Figure 3). The proteins YY-1 and Cyclin Dependent Kinase 6 (CDK6) and Heat-shock protein 47 (HSP47) are regulated in Steps 1 and 4, respectively, arguing a fine-tuning regulation of miR-29a and -b in cervical cancer progress [17,74]. YY1 is a ubiquitously distributed transcription factor involved in repressing and activating a diverse number of promoters. CDK6 is a catalytic subunit of the protein kinase complex that is important for cell cycle G1 phase progression and G1/S transition. HSP47 is localized in the endoplasmic reticulum and plays a role in collagen biosynthesis as a collagen-specific molecular chaperone. Expressions of these genes are up-regulated in cervical cancers promoting migration and invasion. Given the similarity among miR-29a, -b and -c (Figure 3), and the prediction of pictar, the 3p arms could regulate YY-1, CDK6, and HSP47.

## 13. Anti-Oncogenic miR-34 Family Participates in Cervical Cancer

One of the best markers of cancer development is the alteration of p53 expression that conduces to cancer progression by the misregulation of its effectors like miR-34 family. miR-34a forced expression inhibits High Mobility Group Box 1 (HMGB1), NLR Family CARD Domain Containing 5 (NLRC5), Lactate Dehydrogenase A (LDHA), E2F Transcription Factor 3 (E2F3) and increases Retinoic acid-inducible gene-1 (RIG-1) protein expression via 3′UTR. HMGB1 is a nuclear DNA-binding protein that regulates transcription and is involved in organization of DNA. This protein plays a role in several cellular processes, including inflammation, cell differentiation and tumor cell migration. NLRC5 plays a role in cytokine response and antiviral immunity through inhibition of NF-kappa-B activation and negative regulation of type I interferon-signaling pathways. LDHA catalyzes the conversion of l-lactate and Nicotinamide Adenine Dinucleotide (NAD) to pyruvate and Nicotinamide Adenine Dinucleotide (reduced form) (NADH) in the final step of anaerobic glycolysis generating faster organelle duplication. E2F3 is a transcriptional factor that recognizes a specific sequence motif in DNA and interacts directly with the retinoblastoma protein (pRB) to regulate the expression of genes involved in cell cycle. RIG-1 is a putative RNA helicase that is implicated in a number of cellular processes involving RNA binding and alteration of RNA secondary structure and it is involved in viral double-stranded RNA recognition and the regulation of immune response. HMGB1, NLRC5, LDHA, and E2F3 increase metabolism, proliferation, migration and invasion while RIG-1 increases apoptosis, delays cell cycle transition and inhibits Epithelial-mesenchymal transition (EMT) [75,76,77,78,79].

The guide strand of miR-34 family members has five different nt (Figure 1), while the passenger strand has 10 dissimilar nt (Figure 3), therefore it could be argued a differential cellular effect in response to the targets regulated by each member. Interestingly, the 5p strand of miR-34b is classified as * strand; nevertheless, the reads achieved in 21 experiments from both strands of this miRNA vary in 19 reads [20].

In contrast, the effect of 5p and 3p arms of miR-34c seems to be complementary. While 5p arm of miR-34c exclusively inhibited cell proliferation, the 3p arm inhibited proliferation, migration, invasion, and induced apoptosis [6]. During DNA damage, p53 expression induces cell cycle G1 phase progression delay, and if damage could not be repaired at this point, then the apoptosis program is triggered [80,81]. It could be possible that miR-34c-5p expression affects cell cycle G1 phase [82,83,84,85,86] and that miR-34c-3p expression induces apoptosis. Conversely, once pri-miR-34a and pri-miR-34b/c transcription is finished it is not known how one arm is selected against the other. Previously it was shown that p53 regulates the biogenesis of miRNAs through Drosha interaction, promoting the expression of miR-15a/16b, miR-103/107, miR-143/145, miR-203 and miR-206 [87]. Currently, it is not known whether p53 regulates miR-34 family arm selection or what other proteins participate in this process, as it has been shown that miRNA mature expression is tissue specific [11].

## 14. Anti-Oncogenic miR-99 Family Participates in Cervical Cancer

miR-99 family that is composed of miR-99a, miR-99b and miR-100 is intriguingly important because has one of the most ancient miRNAs, miR-100. This miRNA arises from miR-10 family [3]. The expression pattern and validated targets of the members of miR-99 family vary in cervical cancer [17]. miR-99a inhibits protein synthesis of TRIB2 via 3′UTR [88]. miR-100 has shown a gradual expression reduction from Step 1 to 4 of carcinogenesis. Additionally, its expression inversely correlated with the expression of PLK1, a key mitotic checkpoint regulatory protein that is usually highly expressed in Step 4 [89]. Interestingly, PLK1 is regulated at protein but not at mRNA level by miR-100 [90]. Other important targets of miR-100 are phosphatase (CTD (carboxy-terminal domain), RNA polymerase II, polypeptide A small phosphatase-like (CTDSPL), enzyme *N*-Myristoyltransferase 1 (NMT1), Transmembrane Protein 30A (TMEM30A), and chromatin remodeler SWI/SNF Related, Matrix Associated, and Actin Dependent Regulator of Chromatin, Subfamily A, Member 5 (SMARCA5). The three members of miR-99 family show relevance in cervical carcinoma since it has been shown a down-regulation of mRNA and protein of Homeobox A1 (HOXA1) and mTOR via 3′UTR in HaCaT cells. Interestingly, down-regulation of the targets of this family hinders proliferation and migration [91], adding importance of loss of function of miR-99 family in cervical carcinogenesis progression. The targets of miR-100, CTDSPL, TMEM30A and SMARCA5 could be potentially regulated by miR-99a and miR-99b as well (Target Scan, microrna.org, MIRDB and pictar). The 5p strands of the members of miR-99 family have four dissimilar nt (Figure 2), while the 3p strands have seven different nt (Figure 3). Seed sequence of the 3p strand has one different nt between miR-99a/b and miR-100 probably adding different target specificity.

## 15. Anti-Oncogenic miR-124 Family Participates in Cervical Cancer

miR-124 family is formed by miR-124-1, miR-124-2 and miR-124-3. First and second miRNAs are localized in chromosome 8 and the third one in chromosome 20. miR-124-1 is transcribed from the negative DNA strand while miR-124-2 and miR-124-3 are transcribed from the positive strand [20]. Recently it has been shown that miR-124 is down-regulated by DNA methylation in cervical cancer progression showing a higher DNA methylation in Step 3 of cancer progression and cervical cancer than in High-Risk Human Papillomavirus (HR-HPV)-positive tissues [92,93,94] causing Insulin Like Growth Factor Binding Protein 7 (IGFBP7) increase at the mRNA and protein level [92]. IGFBP7 has been implicated in cervical cancer, and it may influence the persistence of HR-HPV infection [95,96]. Restoring miR-124 expression, Angiomotin Like 1 (AmotL1) and Astrocyte-Elevated Gene-1 (AEG-1) are down-regulated via 3′UTR. AmotL1, a peripheral membrane protein that is a component of tight junctions, controls paracellular permeability and maintains cell polarity, while AEG-1 activates the nuclear factor κ-B (NF-κ-B) transcription factor and participates in cytoskeleton remodeling regulation of actin by Rho GTPases. Moreover, miR-124 overexpression inhibits proliferation, migration and invasion [97,98] implying that miR-124 methylation provides advantages for carcinogenesis [92,93,94]. Given the similarity of 5p and 3p strands it could be possible that both strands are selected equally (Figure 2 and Figure 4), however, the 3p strand is selected preferentially [20].

## 16. Anti-Oncogenic miR-125 Family Participates in Cervical Cancer

Family miR-125 is formed by miR-125a, miR-125b-1 and miR-125b-2 localized in chromosomes 19, 11 and 21, respectively. Interestingly, PI3K/AKT signaling-pathway controls cell growth, cell division and apoptosis, and it is regulated positively and negatively by miR-125 family members. In silico analysis predicts that AKT1 and PIK3CD have miRNA binding sites in coding sequence (CDS) and in 5′UTR region for miR-125b-3p* and for miR-125a-3p* (RNA22-HSA). PIK3CD has additional sites for miR-125a-5p and miR-125b-5p (Target Scan). mRNA targets and function for some members of this family have been evaluated. By one side miR-125b inhibits PI3K/AKT pathway through down-regulation of mRNA and protein PIK3CD via 3′UTR binding conducing to protein kinase A (AKT) and mTOR phosphorylation shrink inducing tumor volume growth inhibition [99]. By the other side miR-125b inhibits BAK1 protein synthesis via mRNA 3′UTR binding inhibiting apoptosis and inducing tumor growth volume [100]. miR-125b regulates numerous genes such as oncogenes and tumor suppressors; therefore, the number of genes regulated by this miRNA should determine the effect. Intriguingly, miR-125a-5p shows an increased expression while miR-125b-5p records a decrease from Step 2 to Step 4 of carcinogenesis [17]. The described expression of miR-125a is contrary [101,102] to previously reported [17] while miR-125b continuously has shown similar expression [103]. Expression of miR-125a decreases Signal Transducer and Activator of Transcription 3 (STAT3) and ABL Proto-Oncogene 2, Non-Receptor Tyrosine Kinase (ABL2) protein expression via 3′UTR. STAT3 is a protein that is phosphorylated by the receptor-associated kinases in response to cytokines and growth factors acting as transcription activators in nucleus. ABL2 is non-receptor tyrosine protein kinase and it plays a role in cytoskeletal rearrangements through its C-terminal of F-actin and microtubule-binding sequences. STAT3 and ABL2 augment proliferation, G2/M cell cycle transition, migration, invasion, and tumor growth in cervical cancer [101,102]. 5p strands of these family members have three different nt between them while the 3p strands have seven different nt. An additional difference between 5p strands is that miR-125a-5p has 24 instead of 22 nt that shares with miR-125b-5p 1 and 2 (Figure 2 and Figure 3).

## 17. miR-133 Family Participates in Cervical Cancer

miR-133 family is formed by miR-133a and -b (Figure 2 and Figure 3), as it can be seen the 5p stand is missing for miR-133b [20] (Figure 2). The strand selection, pri-miRNA, pre-miRNA and chromosomal localization suggest unique type of regulation for the members of this family. 5p and 3p strands share 100% of homology (Figure 2 and Figure 3), therefore, both strands could be selected by RISC, although 3p strand is usually selected instead. The fact that 5p strand was not identified or annotated for miR-133b [20] probably be the result of a molecular evolution for cell survival.

In cervical cancer, a miR-133a decrease was shown [104], while for miR-133b contrary expression and function was reported. A gradual increase was achieved from Step 2 to Step 4 of carcinogenesis [27,105] although another study reported a reduction in Step 4 [106]. miR-133a diminish correlate with lymph node metastasis, histological grade and International Federation of Gynecology and Obstetrics (FIGO) state whilst its forced expression prompts apoptosis and hinders proliferation, colony formation, migration, invasion and tumorigenesis in vivo through EGFR 3′UTR mRNA binding restraining AKT-ERK1/2 pathway signaling [104]. On the other hand, miR-133b up-regulation is accompanied by the down-regulation of mammalian sterile 20-like kinase 2 (MST2), cell division control protein 42 homolog (CDC42) and Ras homolog gene family member A (RHOA) at mRNA and protein level. Decreased expression of its targets augments AKT and MAPKs (ERK1 and ERK2) phosphorylation inducing cell proliferation and colony formation in cervical cell lines [105]. Additionally, miR-133b binds to 3′UTR and regulates negatively the expression of Nucleoporin 214 (Nup214). Nup214 is a member of nuclear pore complex forming a gateway that regulates the flow of macromolecules between the nucleus and the cytoplasm. The protein encoded by this gene is localized to the cytoplasmic face of the nuclear pore complex where it is required for proper cell cycle progression, nucleocytoplasmic transport and participates in spindle assembly kinetochore organization and chromosome assembly functioning as oncogene [106]. Interestingly, despite strand 3p similitude the effects between miR-133a and -b are opposed. This is a clear example of the existence of a very specific strand selection mechanism for gene silencing. miRNA strand selection mechanism elucidation is a major subject of study that will widen the understanding of miRNA function regulation.

## 18. Oncogenic miR-146 Family Participates in Cervical Cancer

miR-146 family is formed by miR-146a and miR-146b that are encoded in chromosomes 5 and 10, respectively [20]. The two members of this family show an increased expression in cervical cancer. Importantly, miR-146a records an increase from Step 2 to 4 of carcinogenesis [17]. The overexpression of miR-146a in cervical cancer does not correlate with its function because its forced expression inhibits proliferation, migration and invasion in cell lines [107]. Strands 5p of miR-146 a and b have 2 nt of difference whilst 3p strands have nine dissimilar nt (Figure 2 and Figure 3). It is worth noting that the 3p strands could present differential target regulation.

## 19. Oncogenic miR-181 Family Participates in Cervical Cancer

miR-181 family is formed as follows: miR-181a-1 and miR-181b-1 is clustered in chromosome 1 and miR-181a-2 and miR-181b-2 cluster located in chromosome 9. The miR-181c and miR-181d cluster is located in chromosome 19 and the members differ on their sequence in 2 nt for strands 5p and 11 nucleotides for strands 3p [108]. It should be noted that the 5p strand for miR-181a and -b share identical sequence in contrast to 3p strands with 3 and 2 nt different, respectively (Figure 1 and Figure 3). It has been reported recently that miR-181a confers radiochemo-resistance by diminishing mRNA and proteins of PKC via 3′UTR binding, therefore decreasing caspase 3/7 activity and hindering apoptosis [109,110]. miR-181b down-regulates adenylyl cyclase (AC), restricting cAMP production and promoting cell proliferation and apoptosis diminution [111]. cAMP production is conducive to PKA activation, which induces transcription of smac/Diablo by CREB, which leads to caspase activation [112]. In contrast miR-181a inhibition halts proliferation, cell cycle by p21 and p27 expression as well as apoptosis induction through Bax increase and Bcl-2 decrease. Moreover, PTEN expression is augmented and AKT and FOXO1 phosphorylation diminished [113]. These pathways seem to be regulated by the 5p strand of the members of miR-181 family, in agreement with their similarity and in silico predictions (microrna.org, MIRDB, and Target Scan).

## 20. miR-196 Family Participates in Cervical Cancer

miR-196 family is formed by miR-196a-1, miR-196a-2 and miR-196b that are localized in chromosomes 17, 12 and 7, generated from negative, positive and negative DNA strands, respectively. Interestingly, the 3p strand is not annotated for miR-196a [20] (Figure 3). miR-196a is overexpressed in serum [114], Cervical Intraepithelial Neoplasia (CIN) 2-3 and cervical cancer tissues inducing proliferation and migration, diminishing mRNA and protein of Netrin 4 (NTN4) via 3′UTR. This protein is a member of Netrin family that promotes neurite growth and cell branching [115]. Furthermore, it has been demonstrated that HOXB8, FOXO1 and p27 ^kip1^ are direct targets of miR-196a increasing Cyclin D1 and diminishing p21 mRNAs [116,117]. However, it should be noted that Liu et al., 2015 [116] and Hou et al., 2014 [117] presented different miR-196a expression. The former reported a diminished expression of miR-196a analyzed by microarrays and Reverse Transcription Polymerase Chain Reaction (RT-PCR) comparing normal tissues with HPV-positive cervix and cervical carcinoma tissues [116]. The last one, recordered an increase of miR-196a expression, by RT-PCR, comparing cervical cancer tissues and cell lines with normal cervical tissues. Additionally, a correlation with advanced tumor stage, poor overall and recurrence-free survival and up-regulation of miR-196a in cervical cancer patients was shown [117]. Both works used the same gene to normalize expression but they used different RNA extraction methods and quantities. An additional difference lays in the type of tissues used in the assays, while Hou et al., 2014 [117] used adjacent normal tissues, Liu et al., 2015 [116] used tissues from different patients. This issue has been common for several works as has been reported before [17]. Interestingly, miR-196b is reduced in cervical cancer compared to normal tissue showing an opposed expression to miR-196a [17]. The 5p strands of miR-196a-1, miR-196a-2 and miR-196b differ in 1 nt (Figure 2), whereas 3p strand of miR-196a-1 is not annotated, and the strands of miR-196a-2-3p and miR-196b-3p are totally different (Figure 3).

## 21. miR-200 Family Participates in Cervical Cancer

miR-200 family is formed by miR-200a, miR-200b and miR-200c. The members of this family are localized in chromosome 1 expressing miR-200a and miR-200b while chromosome 12 contains miR-200c [20,118]. miR-200a* shows an increase from Step 2 and miR-200c incorporates overexpression in Step 3 participating in cervical carcinogenesis [17]. miR-200a has been reported overexpressed in serum [71] whereas miR-200b has a controversial reported expression, while Cheng et al. [119] and Cheng et al. reported [120] a down-regulation, Zeng et al. reported an up-regulation [121]. miR-200b mimic reduces migration and it seems that its overexpression inhibits EMT because *E*-cadherin is augmented and vimentin a matrix metalloproteinase-9 is decreased [119] by targeting RhoE directly which binds Guanosine-5’-triphosphate (GTP) but has no GTPase activity, and appears to act as a negative regulator of cytoskeletal organization leading to loss of adhesion, favoring migration and invasion [120]. On the other hand, miR-200b inhibition inhibits cell migration, invasion and tumor growth by targeting Forkhead Box G1 (FoxG1) a transcriptional repressor [121]. It should be noted that miRNAs have hundreds of mRNAs targets that could be regulated at the same time and that probably the sum of all genes regulated give a particular effect affecting cell signaling pathways and cellular processes [18]. Therefore, the dissimilar sequence for every family member should be taken into account. The 5p strands differ in 10 nt in their sequence while, 3p strands differ in 6 nt. miR-200a-5p differs in 5 nt with miR-200b-5p and 7 nt with miR-200c-5p. miR-200a-3p differs in 5 nt with miR-200b-3p and 6 nt with miR-200c-3p. miR-200c-3p has 23 nt instead of 22 (Figure 2 and Figure 3). In general, for these members, they do not have conserved seed sequence, in both strands it varies in 1 nt. In addition to these features, we should consider that ≥1 nt are added in the biogenesis produced by Dicer, and by the activity of other proteins and enzymes [12,15,122,123].

## 22. Conclusions

miRNA expression has been linked to cancer progression [17] by the regulation of several cellular pathways [18]. Notably, miRNA expression has plentiful levels of regulation, including transcription, biogenesis, stability and addition of several modifications increasing variability of targets and regulation of cellular processes [7,9,15,122,124]. Nevertheless, the strand selection, pre-miRNA and genomic context of miRNA families have not been considered in cancer therapy, diagnosis or prognosis. In this work, we point out the importance of strand preference, conservation and expression of miRNAs families associated with cervical cancer. In general, we observed a constant expression of miRNA guide strand from some members of miRNA families, however for miR-10a, miR-15a, miR-16, and miR-200a expression of both guide and star strands was noticed. Importantly, it is unknown the miRNA star strand expression of the rest of the members of the 20 miRNAs families associated with cervical cancer. Percent increase of conservation could have a preserved program of gene regulation leading to guide and star strand expression. miRNA families associated with cervical cancer present a guide strand conservation that varies from 100% to 54.45% while a less star strand conservation for all family members is noticed. Interestingly, miR-9, miR-124 and miR-133 families present 100% of conservation between strands suggesting 50% probability of strand selection, however, for an unclear mechanism it seems that one strand is preferentially selected for degradation by a complex of proteins [125] or by absence of mRNA targets that as well induces miRNA degradation that could contribute to strand survival [126].

5p strand is preferred for miR-9 family while miR-124 and miR-133 families used 3p strand showing 100% conservation. 5p strand of miR-7 family is chosen preferentially and has 100% conservation. miR-10, miR-27 and miR-196 families share more than 95% conservation. miR-146 and miR-181 families have ~90%; miR-23 and miR-125 have 86.36%; and miR-1, miR-19 and miR-99 have 81.81% conservation. miR-17, miR-29 and miR-34 families have ~77% whilst miR-25 and miR-200 families have 72.72%, and finally the less conserved of all the families associated with cervical cancer, miR-15 family has 54.45% of conservation.

In addition to the percentage of conservation, these families present different sizes in nt chain length. In general, mature 5p strands are larger, showing in this review that two miRNAs are 24 nt, 24 miRNAs 23 nt, 35 miRNAs 22 nt and three miRNAs 21 nt long. Regarding 3p strand, it was observed that seven miRNAs are 23 nt, 50 miRNAs 22 nt, six miRNAs 21 nt and four miRNAs 20 nt long. Length size differences could change mRNA target, binding of proteins and factors, and conformational changes in miRISC triggering different mechanisms of regulation. Drosha and Dicer determine the 5p and 3p strands sequence, respectively. It has been reported that 3′ end of 5p strands and 5′ of 3p strands of miRNAs are frequently modified by Dicer, and other proteins [14], however we observed modifications in the 5′ of 5p strands of miR-9, miR-34b, miR-34c, miR-181a-1, miR-181a-2, miR-181b-1, miR-181b-2, miR-181c, miR-181d, miR-200c and miR-106b making the guide strand in some cases bigger and in others smaller. Changes in 5′ and 3′ ends of 5p and 3p strands must be regulated in fine-tuning mode in response to several stimuli raising numerous questions: What proteins, RNAs or molecules regulate these mechanisms? What modifications in proteins that participate in miRNA biogenesis regulate them? What RNA modifications, such as phosphorylation, methylation, base modifications, etc., regulate these mechanisms?

The analysis of the 20 miRNAs families associated with cervical cancer preferentially selects 67 3p and 65 5p strands. Several families miss miRNA strand conservation and use, such as miR-1 family (miR-1-2-5p and miR-206-5p), miR-23 family (miR-23c-5p), miR-133 (miR-133b-5p), miR-7 family (miR-7-3-3p), and miR-196 (miR-196-1-3p). Four and two 5p and 3p strands, respectively, are absent, suggesting 3p strand preference for the 20 miRNAs families associated with cervical cancer. Remarkable 3p and 5p strands are equally conserved through miRNA evolution and used by miRNA families. In this context, from 11 miRNAs families that use 5p strand, six and three are anti-oncogenic and oncogenic, respectively. miR-7, miR-9, miR-15, miR-34, miR-99, and miR-125 families participate in MAPK, PI3K-AKT, Jak/STAT3, and mTOR cell-signaling pathways’ regulation as well as in apoptosis, cell cycle, autophagy, and EMT potentiating cervical carcinoma development. In contrast, miR-17, miR-146, and miR-181 families are involved in DNA repair systems, telomerase activity increase, autophagy, endocytic sorting and traffic of proteins, and Ras-MAPK modulation potentiating cervical carcinogenesis. These cell signals among other proteins contribute to cell process homeostasis that in cervical cancer are misregulated by the strand expression of miRNA families.

In the case of the 9 miRNAs families that select 3p strand, each four are anti-oncogenic and oncogenic. miR-1, miR-23, miR-29, and miR-124 are implicated in glucose metabolism, Notch signaling, cell adhesion, cytoskeleton remodeling, and cell cycle. miR-19, miR-25, miR-27, and miR-200 are associated with EMT, ubiquin ligase complexes, and Golgi transport. It is important to note that miR-10, miR-133 and miR-196 families are not possible to be marked with a particular function because the three families have two mature miRNA sequences with an opposite function (Table 1). However, in general, 10 mature miRNAs families with anti-oncogenic functions are insufficient to avoid tumorigenesis triggered by seven oncogenic mature miRNA families impacting in proliferation, anchorage independent growth, apoptosis, migration, invasion, autophagy, and Warburg effect taken into account the information available until now. Another central point that should be taken in consideration is that miRNA mature strand expression does not always correlate with its function (Table 1). Nevertheless, miRNA signature could be used for diagnosis and prognosis [17], however for therapy, function must be evaluated and the mechanism elucidated before its use. Expression diminish of miR-29 [74] and miR-218 [127] among others is well documented through CIN 1, 2, 3 and cervical cancer [17]. Based on miRNA expression, it is possible to know the stage of cancer progression, making them ideal molecular markers for diagnosis. Up-regulation of miR-155 [21] and miR-196a [114] and down-regulation of miR-26a [128], miR-125a [101], and miR-126 [129] are associated with poor survival of patients with cervical cancer predicting relapse and survival of patients. Regarding miRNA therapy, it should be taken into account that miRNA overexpression or inhibition could present RNAi off-target [130]. Additionally, it was reported that miR-34a overexpression is not functional if it is not phosphorylated [131]. Known and unknown mechanisms have limited miRNA-based therapy, therefore extensive mechanistic studies are needed to understand the complex miRNA biogenesis, availability of RISC complex and miRNAs modifications that are necessary to achieve therapy efficacy. Furthermore, some proteins and RNAs in miRNA strand selection are still unknown, important for possible miRNA based therapy. In contrast, there are numerous miRNAs, both the 5p and 3p strands, with unknown expression and function that could be important in cervical development, which were not included in this work.

In should be noted that pre-miRNA and genomic context are different for all miRNAs families [20]. It seems that the passenger strand has more variability than guide strand generating two hypotheses: (1) it could be argued that passenger strand has more difference in nucleotides between family members than guide strand because it is usually degraded, therefore the selection machinery is not specific; (2) it could be said that sequence difference in passenger and guide strands between family members obeys specific and fine-tuned biogenesis selection to redirect the regulation of diverse specific set of target mRNAs generated by sequence heterogeneity of the passenger strand. miRNA modifications, strand selection and function are a central issue in cervical cancer development. The findings of these issues could contribute to accurate design of miRNA therapy, diagnosis and prognosis approaches.

## Figures and Tables

**Table 1:** miRNAs families showing cervical cancer miRNA expression, function, chromosome localization, DNA strand, mature strand conservation, and nt chain sizes. ? = unknown, + DNA strand = strand direction from 5′ to 3′, − DNA strand= strand direction from 3′ to 5′, C = Conserved strand, NC = Not conserved strand, A = Anti-oncomiR, O = OncomiR, nts = nucleotides.

miRNA Family	Mature Strand	Expression	Function	Chromosomal Localization	DNA Strand	Strand Conservation	nts
miR-1-1	5p	?	?	20	+	NC	22
	3p [22,23]	Down	A			C	22
miR-1-2	3p [22,23]	Down	A	18	−	C	22
miR-206	3p [24]	Down	A	6	+	C	22
miR-7-1	5p [22,23,25,26]	Up/Down	A	9	−	C	22
	3p	?	?			NC	22
miR-7-2	5p [22,23,25,26]	Up/Down	A	15	+	C	22
	3p	?	?			NC	22
miR-7-3	5p [22,23,25,26]	Up/Down	A	19	+	C	22
miR-9-1	5p [27,28,29,30]	Up	A	1	−	C	21
	3p	?	?			NC	22
miR-9-2	5p [27,28,29,30]	Up	A	5	−	C	21
	3p	?	?			NC	22
miR-9-3	5p [27,28,29,30]	Up	A	15	+	C	21
	3p	?	?			NC	22
miR-10a	5p [28,31,32,33]	Up	O	17	−	C	22
	3p [33]	Down	?				22
miR-10b	5p [22,23,34]	Down/Up	A	2	+	C	22
	3p	?	?			NC	22
miR-15a	5p [34,35]	Up	A	13	−	C	22
	3p [36]	Up	A			NC	22
miR-15b	5p [37,38,39,40]	Up	?	3	+	C	22
	3p	?	?			NC	22
miR-16-1	5p [31,34,39,41]	Up	A	13	−	C	22
	3p	?	?			NC	22
miR-16-2	5p [31,34,39,41]	Up	A	3	+	C	22
	3p [42]	Up	?			NC	22
miR-195	5p [22,34,40,43,44]	Down	A	17	−	C	22
	3p	?	?			NC	22
miR-17	5p [28,34,45]	Down	?	13	+	C	22
	3p	?	?			NC	22
miR-18a	5p [46,47]	Up	A	13	+	C	23
	3p	?	?			NC	23
miR-18b	5p	?	?	X	−	C	23
	3p	?	?			NC	23
miR-20a	5p [22,48,49,50,51]	Up	A	13	+	C	22
	3p	?	O			NC	22
miR-20b	5p [22,28,34,35]	Up	?	X	−	C	22
	3p	?	?			NC	22
miR-93	5p [22,34,40,52,53]	Up	O	7	−	C	22
	3p	?	?			NC	22
miR-106a	5p	?	?	X	−	C	23
	3p	?	?			NC	22
miR-106b	5p [34,35,40,54]	Up	O	7	−	C	21
	3p	?	?			NC	22
miR-19a	5p	?	?	13		NC	22
	3p [40,55]	Up	O			C	23
miR-19b-1	5p	?	?	13		NC	23
	3p [40,55]	Up	O			C	23
miR-19b-2	5p	?	?	X		NC	22
	3p [40,55]	Up	O			C	23
miR-23a	5p	?	?	19	−	NC	22
	3p	?	?			C	21
miR-23b	5p	?	?	9	+	NC	22
	3p [40,56,57]	Down	A			C	21
miR-23c	3p	?	?	X	−	C	22
miR-25	5p	?	?	7	−	NC	22
	3p [41,58]	Up	A			C	22
miR-92a-1	5p	?	?	13	+	NC	22
	3p [40,41,43,59,60]	Up	O			C	22
miR-92a-2	5p	?	?	X	−	NC	22
	3p [40,41,43,59,60]	Up	O			C	22
miR-92b	5p	?	?	1	+	NC	22
	3p	?	?			C	22
miR-27a	5p	?	?	19	−	NC	22
	3p [31,40,49,61,62]	Up	O			C	21
miR-27b	5p	?	?	9	+	NC	22
	3p [40,49,63,64]	Up/Down	O			C	21
miR-29a	5p	?	?	7	−	NC	22
	3p [31,41,43]	Down	A			C	22
miR-29b-1	5p	?	?	7	−	NC	22
	3p [65]	Down	?			C	23
miR-29b-2	5p	?	?	1	−	NC	22
	3p [65]	Down	?			C	23
miR-29c	5p	?	?	1	−	NC	22
	3p [31,43]	Down	?			C	22
miR-34a	5p [49,66,67]	Down	A	1	−	C	22
	3p	?	?			NC	22
miR-34b	5p [28,68]	Down	A	11	+	C	23
	3p	?	?			NC	22
miR-34c	5p [28]	Down	A	11	+	C	23
	3p	?	?			NC	22
miR-99a	5p [22,31,34,40,43]	Down	A	21	+	C	22
	3p	?	?			NC	22
miR-99b	5p [22,23]	Down	A	19	+	C	22
	3p	?	?			NC	22
miR-100	5p [22,34,40,41,43,69]	Down	A	11	−	C	22
	3p	?	?			NC	22
miR-124-1	5p	?	?	8	−	NC	22
	3p [70,71,72]	Down	A			C	20
miR-124-2	5p	?	?	8	+	NC	22
	3p [70,71,72]	Down	A			C	20
miR-124-3	5p	?	?	20	+	NC	22
	3p [70,71,72]	Down	A			C	20
miR-125a	5p [37,40,49,73,74]	Up/Down	A	19	+	C	24
	3p	?	?			NC	22
miR-125b-1	5p [23,34,40,49,75]	Down	A	11	−	C	22
	3p	?	?			NC	22
miR-125b-2	5p [23,34,40,49,75]	Down	A	21	+	C	22
	3p	?	?			NC	22
miR-133a-1	5p	?	?	18	−	NC	22
	3p [27,76,77]	Down	A			C	22
miR-133a-2	5p	?	?	20	+	NC	22
	3p [27,76,77]	Down	A			C	22
miR-133b	3p [27,76,78]	Up	O	6	+	C	22
miR-146a	5p [39,40,79]	Up	O	5	+	C	22
	3p	?	?			NC	22
miR-146b	5p [22,34]	Up	?	10	+	C	22
	3p	?	?			NC	22
miR-181a-1	5p [80,81]	Up	O	1	−	C	23
	3p	?	?			NC	22
miR-181a-2	5p [80,81]	Up	O	9	+	C	23
	3p	?	?			NC	22
miR-181b-1	5p [82]	Up	O	1	−	C	23
	3p	?	?			NC	21
miR-181b-2	5p [82]	Up	O	9	+	C	23
	3p	?	?			NC	20
miR-181c	5p	?	?	19	+	C	23
	3p	?	?			NC	22
miR-181d	5p	?	?	19	+	C	23
	3p	?	?			NC	21
miR-196a-1	5p [83,84,85]	Up	O	17	−	C	22
miR-196a-2	5p [83,84,85]	Up	O			C	22
	3p	?	?	12	+	NC	22
miR-196b	5p [57,86]	Down	A	7		C	22
	3p	?	?		−	NC	22
miR-200a	5p [40]	Up	?	1	+	NC	22
	3p [22,52,87]	Up	O			C	22
miR-200b	5p	?	?	1	+	NC	22
	3p [88,89,90]	Up/Down	A			C	22
miR-200c	5p	?	?	12	+	NC	22
	3p [34,37,40,49]	Up/Down	O			C	23
